# Modification of contact avoidance behaviour associated with pyrethroid resistance in *Anopheles sinensis* (Diptera: Culicidae)

**DOI:** 10.1186/s12936-019-2765-3

**Published:** 2019-04-11

**Authors:** Zhengbo He, Jing Zhang, Zongpan Shi, Jingang Liu, Jingjing Zhang, Zhentian Yan, Bin Chen

**Affiliations:** Chongqing Key Laboratory of Vector Insects, Institute of Entomology and Molecular Biology, College of Life Sciences, Chongqing Normal University, Chongqing, 401331 People’s Republic of China

**Keywords:** *Anopheles sinensis*, Behavioral modifications, Resistance, Deltamethrin

## Abstract

**Background:**

*Anopheles sinensis* is the primary vector of vivax malaria in China and its control is under great threat as the development of insecticide resistance. In contrast to physiological resistance, there is no report of behavioural modifications of resistant *An. sinensis* after long-term insecticide use, despite their huge potential impact on malaria transmission.

**Methods:**

Larvae or pupae of *An. sinensis* were collected from Yuanyang, Bishan, and Wuhe counties from southwestern to eastern China. Resistance to deltamethrin was assayed using the standard World Health Organization (WHO) susceptibility test. The frequency distribution of the *kdr* allele of the *para*-type sodium channel gene was determined by polymerase chain reaction (PCR) amplification and DNA sequencing. Contact repellency to deltamethrin-impregnated bed nets was evaluated using a modified WHO cone bioassay.

**Results:**

All contemporary field populations for all three geographic locations were resistant to deltamethrin, with mortality ranging from 6.00 to 26.79%. Three *kdr* genotypes with either an L1014F or L1014C substitution with frequencies of 76.10–100% were identified in the Bishan and Wuhe populations, but no *kdr* mutations were detected in the Yuanyang samples despite high phenotypic resistance. The susceptible mosquitoes exhibited significantly longer flying time and more takeoffs on deltamethrin-treated bed nets (DTN) than on untreated bed nets (UTN), suggestive of robust avoidance behaviour. However, no significant increases in the frequency of takeoffs or flying time were observed in deltamethrin-resistant *An. sinensis* populations when exposed on DTNs, regardless of the presence of a *kdr* mutation. Moreover, the first takeoff from DTNs by resistant mosquitoes significantly lagged behind compared to susceptible mosquitoes.

**Conclusion:**

The *An. sinensis* populations were highly resistant to deltamethrin and exhibited decreased avoidance behaviour. Behavioural modification significantly associated with deltamethrin resistance, but not directly related to the presence of *kdr* mutations, indicating that there are additional factors contributing to the changes.

## Background

Malaria has a high mortality, and has caused nearly a half million deaths in Africa, most of which were in children under 5 years of age [1]. Insecticide-treated mosquito nets (ITNs) and indoor residual spraying (IRS) are widely used for malaria prevention [2]. Organochloride, organophosphate, carbamate, and pyrethroid insecticides are approved for IRS. Only pyrethroids are allowed in ITNs owing to their low mammalian toxicity and high insecticidal potency [3, 4]. Deltamethrin and permethrin are two pyrethroids that have been used to control mosquito vectors in China since the 1980s [5]. ITNs and IRS are effective in reducing vector-human contact [6]. However, extensive use of insecticides has subjected mosquitoes to intensive selection pressure, resulting in the development of physiological resistance and behavioural change [7–9]. Multiple mechanisms contribute to the physiological resistance to pyrethroids in mosquitoes, including target-site insensitivity caused by *kdr* mutations in the *para*-type sodium channel gene (knockdown resistance) and detoxification by mosquito enzymes that metabolize the insecticide before it reaches its target (metabolic resistance) [10].

Insect behavioural responses to insecticides can be stimulus-dependent or independent [11]. Stimulus-independent responses are not associated with the perception of chemicals, but rather involve behaviours like exophily or zoophily that avoid insecticide exposure [8, 11]. Modifications of stimulus-independent behaviours have been observed in malaria vectors following long-term extensive usage of insecticides, including a switch toward outdoor feeding [12–17], change in feeding time [18], increased zoophagy [19, 20] and a shift in malaria vector species [21–24]. Stimulus-dependent behaviours are specific responses to detection of chemicals following contact (irritancy) or without direct contact (repellence) with a treated surface [8, 11]. Insect avoidance behaviour is generally used to describe actions that are stimulus-dependent by some combination of excitation and repellency [11]. A high-throughput excito-repellency assay found that pyrethroids elicited a range of contact irritant and noncontact repellent responses in many mosquito species that typically resulted in avoidance behaviours [11]. Stimulus-dependent behaviours can also be modified by the selection pressure exerted by long-term insecticide use. The avoidance behaviour of pyrethroid-resistant mosquitoes weakens or disappears following contact with pyrethroid-treated bed nets, leading to significantly altered host-seeking behaviours [25–29]. Both stimulus-dependent and stimulus-independent behaviours have strong effects on host contact by aggressive malaria vectors. A change in the response to insecticide-treated surfaces can alter host-seeking behaviour, and thus threaten the effectiveness of indoor vector control tools [8, 29].

*Anopheles sinensis* is a primary vector of vivax malaria in China and other Southeast Asian countries because of its wide distribution and high density [30–32]. The emergence and spread of pyrethroid resistance has been documented in many regions of China, including Hainan [33], Yunnan [34, 35], Chongqing [36], Henan [37], Anhui [35], Shandong [38], Hubei [34], Hunan [34], Jiangsu [39], and Fujian [40] provinces. Resistance has been shown to be associated with mutations at codon L1014 of the *para* sodium channel gene. Four nonsynonymous mutations have been identified, namely, L1014F, L1014S, L1014C, and L1014W [5, 33–35, 38, 41–47]. There have been no reports of behavioural modifications in pyrethroid-resistant *An. sinensis* after long-term use of insecticides. This study examined the level of deltamethrin resistance and *kdr* mutation genotypes in *An. sinensis* from three localities in China. The contact irritancy to permethrin-impregnated nets was assayed in resistant field-collected and susceptible laboratory-raised mosquitoes.

## Methods

### Mosquito strains

**Fig. 1:**
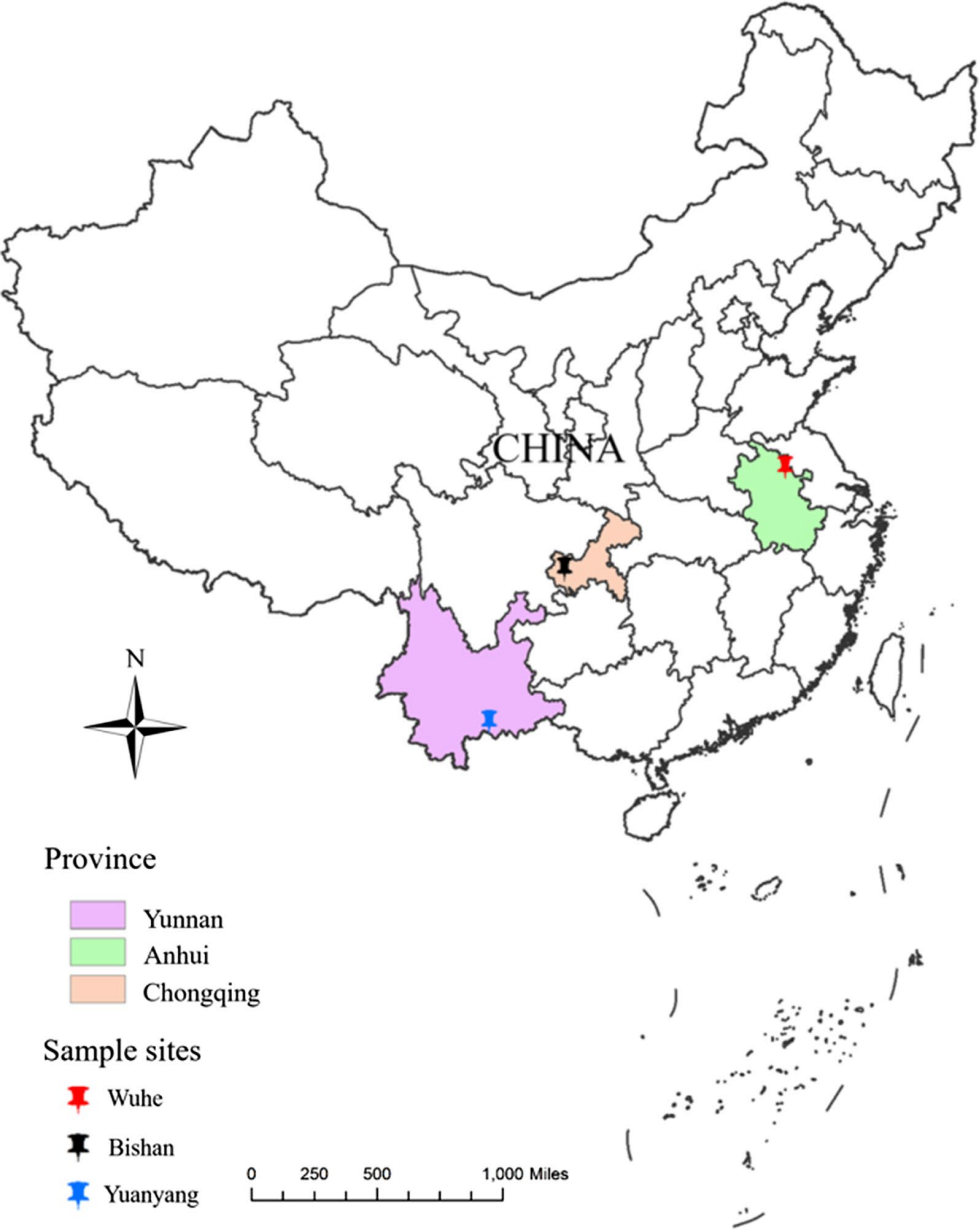
Location of the three mosquito collection sites

Four populations of *An. sinensis*—one laboratory-raised susceptible and three field-collected resistant populations—were used for *kdr* mutation analysis and bioassays. The laboratory population (WX-LS) was obtained from Jiangsu Institute of Parasitic Diseases in Wuxi, China and reared at 27 °C and 80% humidity at the Institute of Entomology and Molecular Biology, Chongqing Normal University. Larvae were fed fish food in clean water and maintained in a 12 h:12 h light:dark photoperiod until pupation. Adults were fed 10% glucose solution. The resistant populations were collected from three geographical sites from southwestern to eastern China, Yuanyang County in Yunnan Province (YY-FR), Bishan District in Chongqing Municipality (BS-FR), and Wuhe County in Anhui Province (WH-FR) (Fig. 1).

Yuanyang is situated in a mountainous area in the south of Yunnan Province close to the border of Vietnam. Bishan is a hilly region located in the west of Chongqing. Wuhe is a county in the north of Anhui Province with many alluvial plains. Rice is the major agricultural crop in the three collection regions with two or three harvests each year in Yuanyang and one harvest in Bishan and Wuhe. Malaria is endemic in Yuanyang and Wuhe, while Bishan is historically endemic [48–50]. Severe damage to the rice crop by insect pests has led to intensive insecticide use with several rounds of spraying in each growing season. Pyrethroids are commonly used for agricultural pest control in the collection sites, but other insecticides such as organic phosphates and carbamates are also used [5, 35].

### Mosquito sampling

About 3000 larvae or pupae from more than ten habitats, most of which were rice fields or small ponds with aquatic plants, were collected at each geographical region. The collection sites were separated by at least 5 km to avoid including genetically-related siblings in the subsequent bioassays. The specimens were identified as *Anopheles* by their morphological features [51] and were kept in plastic storage bins for transport to a local rearing shelter close to the collection site. The larvae and pupae were reared to adults with fish food under local environmental conditions, and the adults were fed 10% glucose solution. Adult females were used in the deltamethrin resistance and behaviour assays and for *kdr* allele genotyping. Mosquitoes were collected and the assays were conducted at the three sampling sites in June–August 2017 and 2018.

### Insecticide susceptibility assay by the WHO standard tube-test method

The susceptibility of adult mosquitoes to deltamethrin was assayed using the standard WHO tube-test method [52]. Twenty-five 3-day-old female mosquitoes were placed in a WHO tube containing 0.05% deltamethrin test paper that was provided by the Chinese Center for Disease Control and Prevention. Paraffin oil-treated paper without insecticide was used as a control. The knockdown time of each mosquito was recorded in minutes up to 1 h. After 1-h exposure to the diagnostic concentration of deltamethrin, mosquitoes were transferred to recovery cups and maintained on 10% glucose solution for 24 h. Surviving and dead mosquitoes were then identified as previously described and counted [34]. Dead mosquitoes were preserved in 95% alcohol in separate containers for subsequent DNA analysis.

### Contact repellency tested by the WHO cone bioassay

Contact repellency was evaluated in adult female mosquitoes by the WHO cone bioassay (WHO/CDS/WHOPES/GCDPP/2005.11). Three-day-old female mosquitoes of field-collected and laboratory populations were used for the test. Mosquitoes were individually introduced into standard WHO cones which were separately placed on a 25 cm × 25 cm net treated with 55 mg/m^2^ ± 25% deltamethrin. Control mosquitoes of the same population were tested on an a net free of insecticide. The irritant effect of deltamethrin were assessed by comparing the time until the first takeoff from the net surface, the number of takeoffs, and the cumulative flying time in a 3-min test time. The mosquitoes were then transferred to individual recovery cups and maintained on 10% glucose solution for 24 h. Surviving and dead mosquitoes were identified and counted as previously described [34]. One leg was removed from each mosquito and preserved individually in 95% alcohol for subsequent species identification and *kdr* mutation analysis.

### DNA extraction and species identification

Individual mosquito legs were homogenized in 50 μl 5% Chelex100 sodium plus 2 μl 20 μg/μl protein K and heated in a water bath for 6 h at 56 °C and at 94 °C for 3 min for extraction of DNA. The extracted DNA solution was used immediately in the PCR assay or stored at − 20 °C for later use. Molecular identification of *An. sinensis* species was done with species-specific primers and amplification of the internal transcribed spacer (ITS) 2 DNA and 28S rDNA regions (D1 and D2) [34]. The PCR mixture contained 7.5 μl of 2× Taq Master Mix (TaKaRa, Dalia, China), 0.8 μl of each primer, 4.9 μl of double distilled H_2_O and 1 μl of the DNA template in a total volume of 15 μl. The PCR was performed at 95 °C for 3 min and 35 cycles of 95 °C for 30 s, 55 °C for 30 s, 72 °C for 30 s, and 72 °C for 10 min. DNA sequencing was carried out using the D1 and D2 *An. sinensis* primers.

### *kdr* gene amplification and sequencing

PCR and direct DNA sequencing were used to identify point mutations of the *kdr* alleles of the *para*-type sodium gene at 1014L in the field-collected mosquitoes as previously described by Zhong et al. [34]. A 325 bp fragment of the *para*-type sodium gene containing position 1014 was amplified using *Kdr*-F (5′-TGCCACTCCGTGTGTTTAGA-3′) and *Kdr*-R (5′-GAGCGATGATGATCCGAAAT-3′) primers for *An. sinensis*. The PCR mixture contained 12.5 μl of 2× Taq Master Mix (TaKaRa, Dalia, China), 1 μl of each primer, 9.5 μl of double distilled H_2_O and 1 μl of the DNA template in a total volume of 25 μl. The PCR reaction was carried out in a Mastercycler ep realplex (Eppendorf; Hamburg, Germany) with initial denaturation at 95 °C for 3 min, followed by 35 cycles of amplification at 95 °C for 30 s, 55 °C for 30 s, and 72 °C for 30 s, with a final extension step at 72 °C for 10 min. The amplicons were resolved in 1% (wt/vol) agarose and observed with UV light. The PCR products were directly sequenced in both forward and reverse directions using the same primers.

### Statistical analysis

Insecticide resistance or susceptibility of the mosquito populations were described by the WHO criteria, with resistance as 80% mortality, probable resistance as 80–98% resistance, and susceptibility as 98% mortality. DNA sequences were translated into amino acid sequences with the EMBL-EBL online tool (http://web.expasy.org/translate/). BioEdit sequence analysis software was used to analyse the mutation. The number of takeoffs, cumulative flying time, and the time until the first takeoff were calculated in each treatment. Statistical analysis was performed with SPSS for Windows 10 (version 19; SPSS Inc., Chicago, IL, USA). For sample characterization, descriptive statistics (mean and standard deviation) were used; *p*-values < 0.05 were considered statistically significant. The Kolmogorov–Smirnov test was performed to examine the distribution of variables. One-way analysis of variance (ANOVA) and the least significant difference multiple comparison test was used for multiple comparison of mortality and knockdown rate. The Chi square statistic test was used to compare the *kdr* mutation frequency, and the non-parametric test (Mann–Whitney U or Kruskal–Wallis test) were used to detect statistically differences for paired or multiple data due to lack of normal distribution. To evaluate the factors that influence the avoidance behaviour of resistant mosquitoes, the Generalized Linear Mixed Model analysis (GLMM) with the logit link function was conducted for number of takeoffs, flying times and the time until first takeoff, respectively. In this models, resistance and *kdr* mutation were established as fixed effects; each tested mosquito was considered as a random effect.

## Results

### Deltamethrin resistance of adult female field-collected *Anopheles sinensis*

The susceptibility of mosquitoes to 0.05% deltamethrin is shown in Table 1. All three tested populations were highly resistant to deltamethrin, with mortality rates between 6.00 and 26.79% and knockdown rates between 9.00 and 36.40%. ANOVA found a significant variation in knockdown (F = 49.14, *p *< 0.0001) and mortality (F = 34.98, *p *< 0.0001), indicating that the WH-FR mosquitoes are the most resistant, followed by YY-FR and BS-FR populations. Mortality was 100% in the laboratory-raised susceptible mosquitoes, confirming the quality of the resistance-test paper.

**Table 1:** Deltamethrin mortality and resistance in a batch of *An. sinensis* in at least three assay replicates

Collection site	Latitude/longitude	Sample size	Knockdown (%)		Mortality (%)		Resistance status
			Mean	95% CI	Mean	95% CI	
Bishan County	N: 29°06′, E: 106°22′	117	36.40^a^	26.64–46.16	26.79^a^	15.35–38.22	R
Yuanyang County	N: 23°04′, E: 102°44′	142	15.21^b^	4.27–26.14	11.07^b^	3.77–18.37	R
Wuhe County	N: 33°15′, E: 117°88′	200	9.00^c^	2.91–15.09	6.00^c^	2.33–9.67	R

CI: confidence interval

^a, b, c^*p *< 0.05 by ANOVA

### Molecular identification

The field-collected mosquitoes were initially identified as belonging to the genus *Anopheles* by their morphology. A total of 1109 adult females collected at the three study sites were tested for deltamethrin susceptibility, contact behaviour, and *kdr* genotype. The 674 that were tested by amplification of ITS2 DNA and 28S rDNA were all identified as *An. sinensis*.

### *kdr* mutations in field-collected *Anopheles sinensis*

**Fig. 2:**
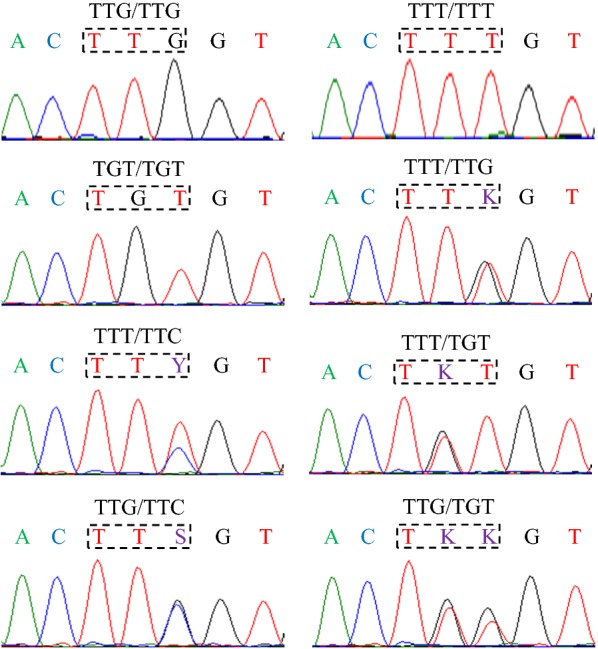
Nucleotide sequence chromatograms of *kdr* genotypes identified in *Anopheles sinensis* from China. The position at codon 1014 of the *para*-type sodium channel gene is indicated by a rectangle. Eight genotypes were identified. Three were homozygous and five were heterozygous (K = G/T; Y = T/C; S = G/C)

*kdr* genotyping was performed in 340 *An. sinensis* mosquitoes, 146 from Bishan, 94 from Yuanyang, and 100 from Wuhe. To genotype the *kdr* alleles, a 325 bp fragment of the *para*-type sodium channel gene at position L1014 (TTG) was amplified and sequenced. No *kdr* mutations were identified in the Yuanyang population despite high levels of phenotypic resistance. Three *kdr* mutations were identified in the Bishan and Wuhe populations. Two L1014F mutations (TTT and TTC) lead to a leucine-to-phenylalanine substitution and one L1014C (TGT) mutation lead to a leucine-to-cysteine substitution. Figure 2 shows the eight genotypes that were identified in the two populations. Three were homozygous (TTG/TTG, TTT/TTT, TGT/TGT) and five were heterozygous (TTT/TTG, TTT/TTC, TTG/TTC, TTT/TGT and TTG/TGT).

### Distribution of *kdr* alleles in field-collected *Anopheles sinensis*

**Fig. 3:**
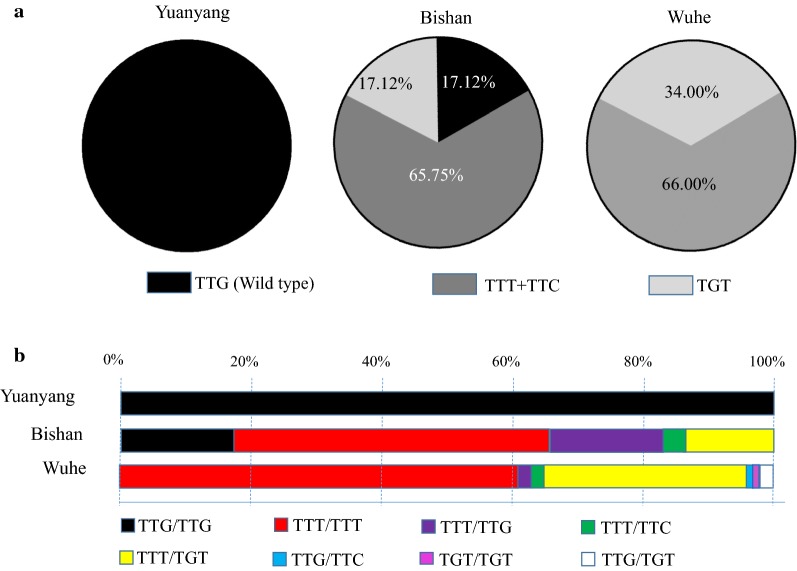
Geographical distribution of *kdr*-L1014 mutations in field-collected *Anopheles sinensis* mosquitoes. **a** Relative frequencies of *kdr*-L1014 mutations. **b** Genotype frequencies of *kdr*-L1014 mutations

*kdr* mutations (Fig. 3a, b) occurred in 82.88% (121/146) of the Bishan mosquitoes, with L1014F (TTT + TTC) accounting for 65.75% (76/146) and L1014C (TGT) accounting for 17.12% (25/146, χ^2^ = 39.370, *p* < 0.001). *kdr* mutations were found in all 100 of the Wuhe mosquitoes. L1014F (TTT + TTC) accounted for 66.00% (66/100) and L1014C (TGT) accounted for 34.00% (34/100, χ^2^ = 20.480, *p* < 0.001).

### Irritability of deltamethrin in susceptible adult female *Anopheles sinensis*

**Fig. 4:**
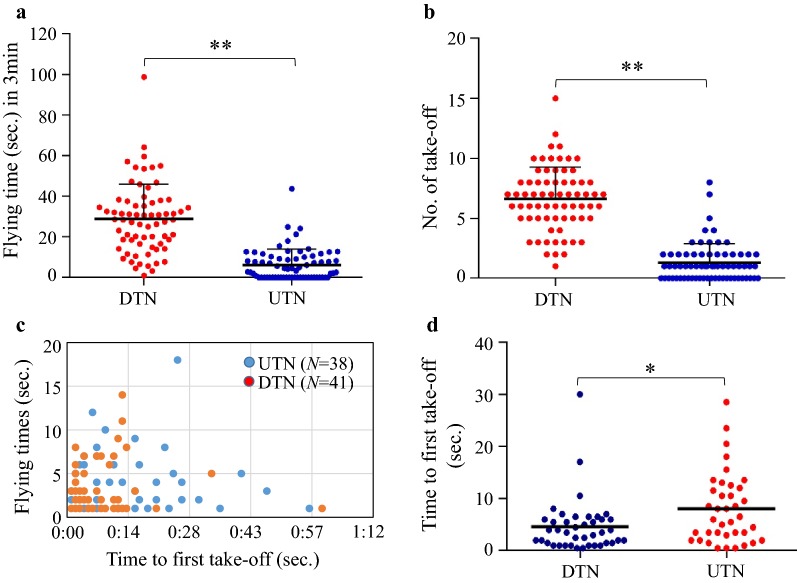
Deltamethrin contact irritancy in susceptible laboratory *Anopheles sinensis* mosquitoes. Flying time (**a**) and number of takeoff (**b**) of female *An. sinensis* during a 3-min exposure to DTNs or UTNs in a modified WHO cone bioassay. **c** The first takeoffs from DTNs occurred early; takeoffs from UTN controls occurred much later in the 3-min interval. Blue dots represent the first takeoffs from UTNs; red dots represent the first takeoffs from DTNs. **d** Time until first takeoff of *An. sinensis* exposed to a DTN or a UTN. N = the number of mosquitoes. Error bars indicate standard errors *p < 0.05, **p < 0.01, Mann–Whitney U test

The irritant effects of deltamethrin on the WX-LS laboratory susceptible population were assessed by comparing the total flying time, number of takeoffs, and time until first take-off over 3 min in a standard WHO cone on deltamethrin-treated (DTN) and untreated (UTN) nets. Total flying time was significantly longer in cones with DTNs (28.84 ± 2.07) than in those with UTNs (6.21 ± 0.94) (Mann–Whitney U test, *z* = − 9.311, *p *< 0.0001). Significantly more takeoffs were observed from DTNs (6.62 ± 0.32) than from UTNs (1.29 ± 0.19) (Mann–Whitney U test, *z *= − 8.389, *p *< 0.0001). The total flying time and number of takeoffs were both significantly increased by exposure to DTN (Fig. 4a, b). First takeoffs occurred sooner from DTNs (8.575 ± 1.087 s) than from UTNs (16.11 ± 3.738 s) (Mann–Whitney U test, *z* = − 2.472, p= 0.013; Fig. 4c, d). DTNs elicited excitation and repellent responses in *An. sinensis* that resulted in moving away from DTNs and avoiding contact with the insecticide.

### Altered irritability in deltamethrin-resistant mosquitoes

**Fig. 5:**
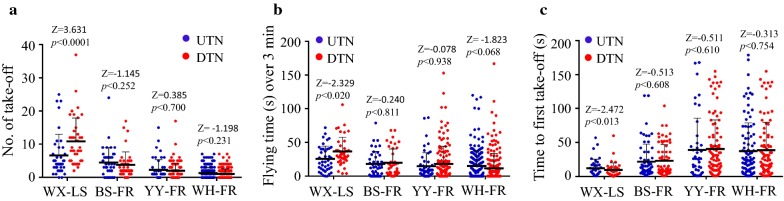
Deltamethrin contact irritancy in the susceptible laboratory (WX-LS) and resistant *Anopheles sinensis* mosquitoes collected at the study regions (BS-FR, YY-FR, and WH-FR). Number of take-off (**a**) and flying time (**b**) of female *An. sinensis* during a 3-min exposure to a DTN or a UTN were determined by a modified WHO cone bioassay. The times to first takeoff from DTNs were compared (**c**). Significances for paired or multiple data were determined by Mann–Whitney U or Kruskal–Wallis test due to lack of normal distribution. Error bars indicate standard errors

The irritabilities of susceptible WX-LS and field-collected resistant mosquitoes were compared using the cone assay described above. The WX-LS mosquitoes made a mean of 6.60 takeoffs from the UTNs and 10.88 from the DTNs. The resistant mosquitos made fewer takeoff than the WX-LS mosquitoes, from 1.30 to 4.49 from the UTNs and from 1.05 to 3.70 from the DTNs (Kruskal–Wallis test: UTNs, df = 3, χ^2^ = 71.65, *p* < 0.0001; DTNs, df = 3, χ^2^ = 139.35, *p* < 0.0001) (Fig. 5a). The mean flying times of WX-LS mosquitoes in cones were 25.60 s for UTNs and 36.54 s for DTNs, while the flying times of resistant mosquitos, from 14.55 to 18.07 s in cones with UTNs and from 11.26 to 19.51 s in cones with DTNs, were shorter than the WX-LS mosquitoes (Kruskal–Wallis test: UTNs, df = 3, χ^2^ = 23.76, *p* < 0.0001; DTNs, df = 3, χ^2^ = 76.93, *p* < 0.0001). The first takeoff averagely occurred at 9.29 s for DTNs and 16.05 s for UTNs in the WX-LS mosquitoes, and at 21.60 to 39.02 s for DTNs and 23.14 to 40.37 s for UTNs in the three resistant mosquitoes. Clearly, the first takeoff occurred significantly later in the resistant populations than it did in the WX-LS mosquitoes on both UTNs (Kruskal–Wallis test, df = 3, χ^2^ = 15.00, *p* = 0.002) and DTNs (Kruskal–Wallis test, df = 3, χ^2^ = 27.32, *p* < 0.0001). The results indicate that the irritability of resistant mosquitoes was significantly decreased relative to the susceptible laboratory mosquitoes.

Generalized Linear Mixed Model analysis with logit link function were used to analyze the causes of behavioural modification. The coefficients for the fixed effects in the model are shown in Table 2, demonstrating that *kdr* mutation was not significantly associated with the decreased in number of takeoffs (*p* = 0.993) or flying times (*p* = 0.428), and not significantly associated with the increase of time until first takeoff (*p* = 0.551) in the resistant mosquitoes. This is consistent with the observation that the irritability of YY-FR mosquitoes, which no *kdr* mutations were identified, was significantly decreased, comparing to the susceptible WX-LS mosquitoes (Fig. 5). However, as shown in Table 2, the resistance level had a significant negative effect on the number of takeoffs and flying times, and had a significant positive effect on time until first takeoff. Among the three resistant populations, WH-FR mosquitoes had the strongest resistance, the largest coefficient and the weakest avoidance behaviour, while BS-FR mosquitoes had the weakest resistance, the smallest coefficient and the strongest avoidance behaviour. The results indicate that the modification of contact avoidance behaviour associated with deltamethrin resistance, but not directly related to *kdr* mutation.

**Table 2:** Parameter estimates, standard error, *t* and associated *p* value of fixed parameters in GLMMs

Model	Fixed effects	Coefficient	Std error	*t*	Sig.	95% CI for coefficient	
						Lower	Upper
No. of takeoffs	Intercept	1.981	0.306	6.466	< 0.0001	1.379	2.582
	Resistance level^a^						
	WH-FR	− 1.953	0.310	− 6.308	< 0.0001	− 2.561	− 1.345
	YY-FR	− 1.420	0.124	− 11.493	< 0.0001	− 1.663	− 1.178
	BS-FR	− 0.774	0.281	− 2.757	0.006	− 1.325	− 0.223
	WX-LS^a^						
	kdr mutation						
	*kdr*-MU	0.003	0.288	0.009	0.993	− 0.562	0.567
	*kdr*-WT^a^						
Total flying times	Intercept	3.230	0.216	14.972	< 0.0001	2.806	3.653
	Resistance level						
	WH-FR	− 2.543	0.617	− 4.124	< 0.0001	− 3.754	− 1.333
	YY-FR	− 1.379	0.246	− 5.603	< 0.0001	− 1.863	− 0.896
	BS-FR	− 1.334	0.565	− 2.362	0.018	− 2.443	− 0.225
	WX-LS^a^						
	kdr mutation						
	*kdr*-MU	0.455	0.573	0.793	0.428	− 0.671	1.580
	*kdr*-WT^a^						
Time until first takeoff	Intercept	12.724	9.733	1.307	0.192	− 6.395	31.843
	Resistance level						
	WH-FR	30.266	11.718	2.583	0.010	7.248	53.285
	YY-FR	28.039	6.230	4.501	< 0.0001	15.802	40.277
	BS-FR	15.066	8.533	1.766	0.078	− 1.696	31.829
	WX-LS^a^						
	kdr mutation						
	*kdr*-MU	− 4.789	8.023	− 0.597	0.551	− 20.550	10.972
	*kdr*-WT^a^						

*kdr*-MU: *kdr* mutated; *kdr*-WT: *kdr* wild type; CI: confidence interval

^a^Denotes the reference category

## Discussion

The deltamethrin resistance was assayed in *An. sinensis* populations from Yuanyang County, Bishan District, and Wuhe County in China. All three field-collected populations had developed high resistance to deltamethrin (Table 1). Three *kdr* genotypes with high frequencies (76.10–100%) of either an L1014F or L1014C substitution were identified in the Bishan and Wuhe populations. No *kdr* mutations were identified in the 94 samples from Yunnan despite a high level of phenotypic resistance, which is consistent with previous reports that pyrethroid resistance in that population was conferred mainly by metabolic mechanisms [34–45]. The development of resistance by *An. sinensis* is consistent with reductions in mortality from 80.72% [53], to 32.72% [36], and 26.79% in Bishan; from 29.6% [34] to 11.07% in Yuanyang, and from 30% [35] to 6.00% in Wuhe. The frequency of *kdr* mutations has increased from 7.23% [36] to 82.88% in Bishan and from 98.4% [35] to 100% in Anhui. The results clearly indicate that deltamethrin resistance has greatly increased in *An. sinensis* in China in recent decades.

The contact irritancy of deltamethrin to *An. sinensis* was tested with a modified WHO cone assay. The results demonstrated that the susceptible mosquitoes were effectively repelled by deltamethrin-impregnated bed nets, resulting in significant avoidance behaviour (Fig. 4). However, the avoidance behaviour of the resistant field-collected mosquitoes was significantly attenuated or even lost in the response to DTNs compared with the susceptible population (Fig. 5). This is the first study to report modified behaviours in resistant *An. sinensis* populations exposed to long-term insecticide selection pressure. Similarly, altered avoidance behaviour has been described in many other pyrethroid-resistant mosquitoes following contact with pyrethroid-treated bed nets [25–29].

Mosquitoes that have become insensitive to pyrethroids after long exposure are not effectively repelled and might remain on treated materials for extended periods, which would provide more opportunities to enter bed nets or stay indoors. Therefore, altered avoidance behaviour would appear to impair the effectiveness of ITNs and IRS. However, previous studies showed that insecticide-treated bed nets remained effective because the decreased avoidance behaviour of resistant mosquitoes resulted in an increased dose of insecticide that resulted in higher mortality [25, 26]. It is thus not clear whether altered avoidance behaviour affects the effectiveness of ITNs and IRS.

The underlying cause of the change in avoidance behaviour remains unclear so far. Point mutations in voltage-gated sodium channels could enhance closed-state inactivation of nerves to interfere with pyrethroid sensitivity, reduce irritancy, and result in slowed avoidance behaviour responses or reduced repellency [54–56]. Chandre et al. [26] reported lost contact repellency to 1% permethrin impregnated paper in a laboratory-selected *Anopheles gambiae* sensu stricto (s.s.) colony originating from Burkina Faso that carried a homozygous (RR) *kdr* allele. Corbel et al. [57] showed that increased insecticide exposure in heterozygous RS *An. gambiae* that stayed longer than susceptible mosquitoes on permethrin-treated surfaces resulted in increased killing. Kawada et al. [27] reported that pyrethroid-resistant *An. gambiae* s.s. with a high allelic frequency of *kdr* (L1014S) lost repellency to pyrethroids, whereas resistant *Anopheles arabiensis* and *Anopheles funestus* s.s. colonies lacking *kdr* mutations retained high repellency even though they possessed metabolic resistance to pyrethroids. Similar responses have been seen in *kdr*-resistant *Heliothis virescens* moths [54] and *Kdr*-mutated *Musca domestica* houseflies [58]. In this study, pyrethroid repellency was not observed in the deltamethrin-resistant *An. sinensis* from Yuanyang County (Fig. 5) despite a lack of *kdr* mutations (Fig. 3). The GLMM analysis also supported that the behavioural modification was not directly related to the presence of *kdr* mutations (Table 2). Thus, it can be seen that the mechanisms underlying the observed behavioural modifications seem to be complex, including factors other than *kdr* that can influence changes in avoidance of insecticides.

The behavioural avoidance of pyrethroids suggests that pyrethroid-impregnated bed nets or sprayed surfaces can be detected by malaria vectors by contact and/or olfaction. Olfaction is active in finding food, hosts, mates and oviposition sites, avoiding predators, pathogens, and harmful chemicals [59]. Pyrethroids have low vapor pressures, but pyrethroid molecules can be detected in indoor air samples as far as 1 m away from cyfluthrin treated nets, indicating that they might be present in the air in spite of their low volatility [60, 61]. Porciani et al. [29] reported that *kdr*-resistant *An. gambiae* mosquitoes were more strongly attracted by host odors emanating behind permethrin-treated nets than by those from behind an untreated net. They perceived the difference of the ITNs and UTNs at a distance and behaved differently in response. The mosquitoes may have detected chemicals released by bed nets with olfactory receptors tuned to respond to them. Bohbot et al. [62] showed that AaOR2 odorant receptor was activated and AaOR8 receptor was inhibited by pyrethroids in the dengue and zika vector *Aedes aegypti*. These results suggested that pyrethroids most likely modulate the function of mosquito ORs by multiple molecular mechanisms to influence olfactory-driven behaviour. Suppression of the *AsOR7* coreceptor in *An. sinensis* by RNA interference also resulted in decreased avoidance behaviour, as seen in resistant mosquitoes (unpublished data). These pioneering studies provide insight into the relationship of the olfactory system and behaviour changes and warrant further research to test this hypothesis.

## Conclusions

All three field-collected mosquito populations were highly resistant to deltamethrin, and their avoidance behaviours were significantly decreased compared with the susceptible population. To the best of our knowledge, this is the first report of behavioural modifications in resistant *An. sinensis* exposed to long-term insecticide selection pressure. The behavioural modifications seem not directly related to the presence of *kdr* mutations. The mechanisms underlying the behavioural modifications are complex and include additional factors that can also lead to changes in behaviour. It is not clear whether changes in avoidance behaviour act as an ecological obstacle to limit the effectiveness of vector control. A better understanding of the behavioural ecology of malaria vectors will help to develop tailor-made vector control strategies, for example, using evolved spatial repellents [63] or chemosterilant pyriproxyfen [64] to generate sustainable control effects.

## Abbreviations

ANOVA: one-way analysis of variance
DTN: deltamethrin-treated bed nets
GLMM: Generalized Linear Mixed Model analysis
IRS: indoor residual spraying
ITNs: insecticide-treated mosquito nets
OR: odorant receptor
PCR: polymerase chain reaction
UTN: untreated bed nets
WHO: World Health Organization

## References

[1] WHO (2017). Global vector control response 2017–2030.

[2] WHO (2006). Indoor residual spraying. Use of indoor residual spraying for scaling up global malaria control and elimination.

[3] Zaim M, Aitio A, Nakashima N (2000). Safety of pyrethroid-treated mosquito nets. Med Vet Entomol.

[4] Zaim M, Jambulingam P (2007). Global insecticide use for vector-borne disease control.

[5] Cui F, Raymond M, Qiao C (2006). Insecticide resistance in vector mosquitoes in China. Pest Manag Sci.

[6] WHO (2016). World malaria report 2016.

[7] Ranson H, N’guessan R, Lines J, Moiroux N, Nkuni Z, Corbel V (2011). Pyrethroid resistance in African anopheline mosquitoes: what are the implications for malaria control?. Trends Parasitol.

[8] Gatton ML, Chitnis N, Churcher T, Donnelly MJ, Ghani AC, Godfray HC (2013). The importance of mosquito behavioural adaptations to malaria control in Africa. Evolution.

[9] Sougoufara S, Doucouré S, Backé Sembéne PM, Harry M, Sokhna C (2017). Challenges for malaria vector control in sub-Saharan Africa: resistance and behavioral adaptations in *Anopheles* populations. J Vector Borne Dis.

[10] Hemingway J, Ranson H (2000). Insecticide resistance in insect vectors of human disease. Annu Rev Entomol.

[11] Chareonviriyaphap T, Bangs MJ, Suwonkerd W, Kongmee M, Corbel V, Ngoen-Klan R (2013). Review of insecticide resistance and behavioral avoidance of vectors of human diseases in Thailand. Parasit Vectors.

[12] Reddy MR, Overgaard HJ, Abaga S, Reddy VP, Caccone A, Kiszewski AE (2011). Outdoor host seeking behaviour of *Anopheles gambiae* mosquitoes following initiation of malaria vector control on Bioko Island, Equatorial Guinea. Malar J.

[13] Russell TL, Govella NJ, Azizi S, Drakeley CJ, Kachur SP, Killeen GF (2011). Increased proportions of outdoor feeding among residual malaria vector populations following increased use of insecticide-treated nets in rural Tanzania. Malar J.

[14] Russell TL, Beebe NW, Bugoro H, Apairamo A, Chow WK, Cooper RD (2016). Frequent blood feeding enables insecticide-treated nets to reduce transmission by mosquitoes that bite predominately outdoors. Malar J.

[15] Moiroux N, Gomez MB, Pennetier C, Elanga E, Djènontin A, Chandre F (2012). Changes in *Anopheles funestus* biting behavior following universal coverage of long-lasting insecticidal nets in Benin. J Infect Dis.

[16] Overgaard HJ, Reddy VP, Abaga S, Matias A, Reddy MR, Kulkarni V (2012). Malaria transmission after five years of vector control on Bioko Island, Equatorial Guinea. Parasit Vectors.

[17] Meyers JI, Pathikonda S, Popkin-Hall ZR, Medeiros MC, Fuseini G, Matias A (2016). Increasing outdoor host-seeking in *Anopheles gambiae* over 6 years of vector control on Bioko Island. Malar J.

[18] Reimer LJ, Thomsen EK, Koimbu G, Keven JB, Mueller I, Siba PM (2016). Malaria transmission dynamics surrounding the first nationwide long-lasting insecticidal net distribution in Papua New Guinea. Malar J.

[19] Lefèvre T, Gouagna LC, Dabiré KR, Elguero E, Fontenille D, Renaud F (2009). Beyond nature and nurture: phenotypic plasticity in blood-feeding behavior of *Anopheles gambiae* s.s. when humans are not readily accessible. Am J Trop Med Hyg.

[20] Waite JL, Swain S, Lynch PA, Sharma SK, Haque MA, Montgomery J (2017). Increasing the potential for malaria elimination by targeting zoophilic vectors. Sci Rep.

[21] Bayoh MN, Mathias DK, Odiere MR, Mutuku FM, Kamau L, Gimnig JE (2010). *Anopheles gambiae*: historical population decline associated with regional distribution of insecticide-treated bed nets in western Nyanza Province, Kenya. Malar J.

[22] Derua YA, Alifrangis M, Hosea KM, Meyrowitsch DW, Magesa SM, Pedersen EM (2012). Change in composition of the *Anopheles gambiae* complex and its possible implications for the transmission of malaria and lymphatic filariasis in north-eastern Tanzania. Malar J.

[23] Mwangangi JM, Mbogo CM, Orindi BO, Muturi EJ, Midega JT, Nzovu J (2013). Shifts in malaria vector species composition and transmission dynamics along the Kenyan coast over the past 20 years. Malar J.

[24] Sougoufara S, Harry M, Doucouré S, Sembène PM, Sokhna C (2016). Shift in species composition in the *Anopheles gambiae* complex after implementation of long-lasting insecticidal nets in Dielmo, Senegal. Med Vet Entomol.

[25] Darriet F, N’Guessan R, Koffi AA, Konan L, Doannio JM, Chandre F (2000). Impact of pyrethrin resistance on the efficacy of impregnated mosquito nets in the prevention of malaria: results of tests in experimental cases with deltamethrin SC. Bull Soc Pathol Exot.

[26] Chandre F, Darriet F, Duchon S, Finot L, Manguin S, Carnevale P (2000). Modifications of pyrethroid effects associated with *kdr* mutation in *Anopheles gambiae*. Med Vet Entomol.

[27] Kawada H, Ohashi K, Dida GO, Sonye G, Njenga SM, Mwandawiro C (2014). Insecticidal and repellent activities of pyrethroids to the three major pyrethroid-resistant malaria vectors in western Kenya. Parasit Vectors.

[28] Diop MM, Moiroux N, Chandre F, Martin-Herrou H, Milesi P, Boussari O (2015). Behavioral cost & overdominance in *Anopheles gambiae*. PLoS ONE.

[29] Porciani A, Diop M, Moiroux N, Kadoke-Lambi T, Cohuet A, Chandre F (2017). Influence of pyrethroïd-treated bed nets on host seeking behaviors of *Anopheles gambiae* s.s. carrying the *kdr* allele. PLoS ONE.

[30] Foley DH, Klein TA, Kim HC, Sames WJ, Wilkerson RC, Rueda LM (2009). Geographic distribution and ecology of potential malaria vectors in the Republic of Korea. J Med Entomol.

[31] Sinka ME, Bangs MJ, Manguin S, Chareonviriyaphap T, Patil AP, Temperley WH (2011). The dominant *Anopheles* vectors of human malaria in the Asia-Pacific region: occurrence data, distribution maps and bionomic précis. Parasit Vectors.

[32] Pan JY, Zhou SS, Zheng X, Huang F, Wang DQ, Shen YZ (2012). Vector capacity of *Anopheles sinensis* in malaria outbreak areas of central China. Parasit Vectors.

[33] Sun DW, Wang GZ, Zeng LH, Li SG, He CH, Hu XM (2017). Extensive resistance of *Anopheles sinensis* to insecticides in malaria-endemic areas of Hainan Province, China. Am J Trop Med Hyg.

[34] Zhong D, Chang X, Zhou G, He Z, Fu F, Yan Z (2013). Relationship between knockdown resistance, metabolic detoxification and organismal resistance to pyrethroids in *Anopheles sinensis*. PLoS ONE.

[35] Fu F, Chen B, Zhong D, Meng F, Yan Z, He Z (2013). The association between deltamethrin resistance and *kdr* mutation in *Anopheles sinensis* in Chongqing, China. Acta Parasitol Med Entomol Sin.

[36] Chang X, Zhong D, Fang Q, Hartsel J, Zhou G, Shi L (2014). Multiple resistances and complex mechanisms of *Anopheles sinensis* mosquito: a major obstacle to mosquito-borne diseases control and elimination in China. PLoS Negl Trop Dis.

[37] Qi X, Cui J (2012). Investigation on the resistance of *Anopheles sinensis* to deltamethrin and its association with the *kdr* genotypes in Yunnan province and parts of Henan province. Chin J Vector Biol Contr.

[38] Dai Y, Huang X, Cheng P, Liu L, Wang H, Wang H (2015). Development of insecticide resistance in malaria vector *Anopheles sinensis* populations from Shandong province in China. Malar J.

[39] Li J, Zhou H, Cao G, Wang W, Gu Y, Liu Y (2011). Sensitivity of *Anopheles sinensis* to insecticides in Jiangsu Province. Chin J Schisto Control.

[40] Wu J, He X, Liu Q (1994). Study of the insecticide sensitivity of malaria vectors in northwest area of Fujian Province. Chin J Vector Biol Control.

[41] Kang S, Jung J, Lee S, Hwang H, Kim W (2012). The polymorphism and the geographical distribution of the knockdown resistance (*kdr*) of *Anopheles sinensis* in the Republic of Korea. Malar J.

[42] Tan WL, Li CX, Wang ZM, Liu MD, Dong YD, Feng XY (2012). First detection of multiple knockdown resistance (*kdr*)-like mutations in voltage-gated sodium channel using three new genotyping methods in *Anopheles sinensis* from Guangxi Province, China. J Med Entomol.

[43] Tan WL, Wang ZM, Li CX, Chu HL, Xu Y, Dong YD (2012). First report on co-occurrence knockdown resistance mutations and susceptibility to beta-cypermethrin in *Anopheles sinensis* from Jiangsu Province, China. PLoS ONE.

[44] Wang DQ, Xia ZG, Zhou SS, Zhou XN, Wang RB, Zhang QF (2013). A potential threat to malaria elimination: extensive deltamethrin and DDT resistance to *Anopheles sinensis* from the malaria-endemic areas in China. Malar J.

[45] Wang Y, Yu W, Shi H, Yang Z, Xu J, Ma Y (2015). Historical survey of the *kdr* mutations in the populations of *Anopheles sinensis* in China in 1996–2014. Malar J.

[46] Zhang HW, Liu Y, Hu T, Zhou RM, Chen JS, Qian D (2015). Knockdown resistance of *Anopheles sinensis* in Henan province, China. Malar J.

[47] Yang C, Feng X, Huang Z, Li M, Qiu X (2016). Diversity and frequency of *kdr* mutations within *Anopheles sinensis* populations from Guangxi, China. Malar J.

[48] Bi Y, Hu W, Yang H, Zhou XN, Yu W, Guo Y (2013). Spatial patterns of malaria reported deaths in Yunnan Province, China. Am J Trop Med Hyg.

[49] Lu G, Zhou S, Horstick O, Wang X, Liu Y, Müller O (2014). Malaria outbreaks in China (1990–2013): a systematic review. Malar J.

[50] Huang JX, Xia ZG, Zhou SS, Pu XJ, Hu MG, Huang DC (2015). Spatio-temporal analysis of malaria vectors in national malaria surveillance sites in China. Parasit Vectors.

[51] Dong X (2010). The mosquito fauna of Yunnan.

[52] WHO (1998). Report of the WHO informal consultation. Test procedures for insecticide resistance monitoring in malaria vectors, bio-efficacy and persistence of insecticides on treated surfaces.

[53] Jiang S, Lei X, Xia R, Chen H, Pu H, Xiao B (1993). Resistance of *Anopheles sinensis* to insecticides in Chongqing. Mod Prevent Med.

[54] Lee D, Park Y, Brown TM, Adams ME (1999). Altered properties of neuronal sodium channels associated with genetic resistance to pyrethroids. Mol Pharmacol.

[55] Kawada H, Clark J, Bloomquist JR, Kawada H (2009). An inconvenient truth of pyrethroid—does it have a promising future?. Advances in human vector control (ACS symposium book 1014).

[56] Vais H, Williamson MS, Goodson SJ, Devonshire AL, Warmke JW, Usherwood PN (2000). Activation of *Drosophila* sodium channels promotes modification by deltamethrin Reductions in affinity caused by knock-down resistance mutations. J Gen Physiol.

[57] Corbel V, Chandre F, Brengues C, Akogbéto M, Lardeux F, Hougard JM (2004). Dosage-dependent effects of permethrin-treated nets on the behaviour of *Anopheles gambiae* and the selection of pyrethroid resistance. Malar J.

[58] Virgona CT, Holan G, Shipp E (1983). Repellency of insecticides to resistant strains of housefly. Entomol Exp Appl.

[59] Andersson MN, Löfstedt C, Newcomb RD (2015). Insect olfaction and the evolution of receptor tuning. Front Ecol Evol.

[60] Bomann W (1995). How safe are pyrethroid-treated mosquito nets? An evaluation based on the example of Solfac EW 050. Bayer Public Health.

[61] Bouvier G, Blanchard O, Momas I, Seta N (2006). Pesticide exposure of non-occupationally exposed subjects compared to some occupational exposure: a French pilot study. Sci Total Environ.

[62] Bohbot JD, Fu L, Le TC, Chauhan KR, Cantrell CL, Dickens JC (2011). Multiple activities of insect repellents on odorant receptors in mosquitoes. Med Vet Entomol.

[63] Lynch PA, Boots M (2016). Using evolution to generate sustainable malaria control with spatial repellents. eLife.

[64] White MT, Lwetoijera D, Marshall J, Caron-Lormier G, Bohan DA, Denholm I (2014). Negative cross resistance mediated by co-treated bed nets: a potential means of restoring pyrethroid-susceptibility to malaria vectors. PLoS ONE.

